# Evaluation of prescribing patterns of sodium valproate in neurological disease patients: a quasi-experimental pretest–posttest design study

**DOI:** 10.1186/s12913-025-12483-5

**Published:** 2025-03-27

**Authors:** Rehab H. Werida, Salima El-Sherif, Rania Shoshan, Naglaa F. Khedr

**Affiliations:** 1 Clinical Pharmacy and Pharmacy Practice, Faculty of Pharmacy, Damanhour University, Damanhour, 22514 P.O Egypt; 2 Faculty of Pharmacy, Mansoura University, Mansoura, Egypt; 3 Neurology Department, Mansoura University Hospital, Mansoura, Egypt; 4 Biochemistry Department, Faculty of Pharmacy, Tanta University, Tanta, Egypt

**Keywords:** Prescribing Pattern, Pharmacy education, Sodium valproate, Anti-seizure, Outpatients, Adherence

## Abstract

**Background:**

The well-known anti-seizure medication, sodium valproate, is used to treat epilepsy, bipolar disorder, and other mental health conditions.

**Objective:**

This study aimed to determine how pharmacist counseling intervention affected the prescribing patterns and usage of sodium valproate in patients with neurological diseases.

**Methods:**

Patient prescriptions were analyzed in a quasi-experimental pretest–posttest design research, at baseline and after three months of pharmacy educational intervention. Medical history, drug-drug interactions, antipsychotic combinations, medication errors, and dosages were among the information gathered.

**Results:**

The reviewed prescriptions observed trend towards antipsychotic combination, 15 (2.5%) prescriptions had five medications, while 18 (3%) contained four, 169 (28.2%) containing three, 329 (54.8%) containing two, and 69 (11.5%) containing one, antipsychotic. Of the reviewed prescriptions, 6% had possible drug-drug interactions. However, following a 3-month pharmacy educational intervention, the tendency toward antipsychotic combination decreased significantly to 262 (52%) with prescriptions for 2 antipsychotics and 163 (32.3%) with 3 antipsychotics. Likewise, the decreases in medication errors related to frequency and dosage were 8 (1.6%) *vs.* 29 (4.8%) and 6 (1.2%) *vs.* 35 (5.8%), compared to baseline respectively. Additionally, medication adherence was raised significantly (*p* < 0.000) from 50 (8.3%) to 338 (67.1%) after the intervention.

**Conclusion:**

More precise regulations should be placed on sodium valproate prescription patterns and usage, throughout implemented pharmacist initiative in patients counselling, provision of health education and therapeutic monitoring to improve health-related quality of life.

**Trial registration:**

This study was registered on clinicaltrial.gov with an identification code NCT05830981. https://classic.clinicaltrials.gov/ct2/show/NCT05830981 (First Posted: April 26, 2023).

**Graphical abstract:**

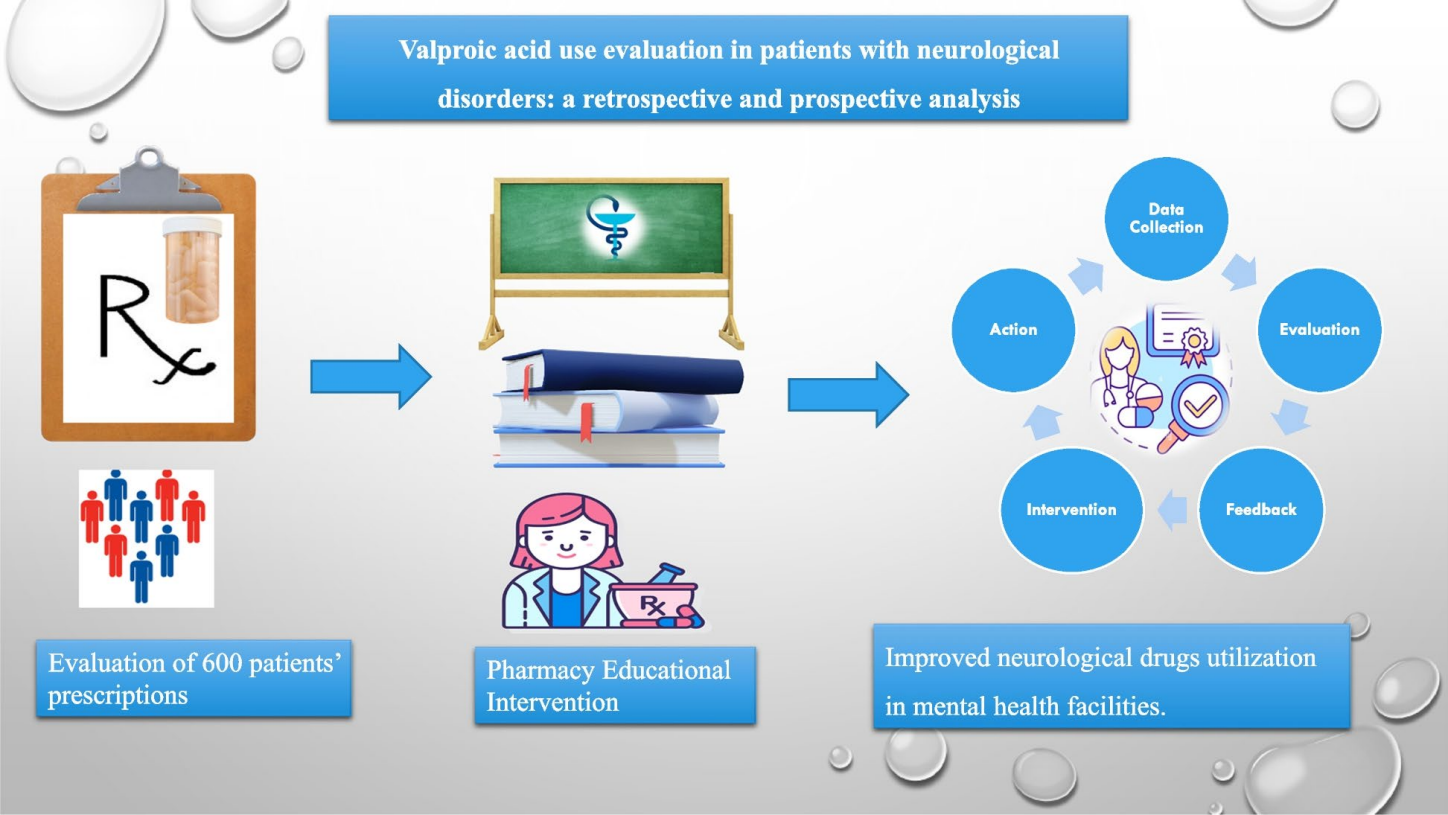

**Supplementary Information:**

The online version contains supplementary material available at 10.1186/s12913-025-12483-5.

## Key points

• Sodium valproate is used to treat neurological diseases.

• For individuals with neurological disorders, routine pharmacist assessments of medication regimens are crucial to identifying and managing any possible drug-related issues.

• In patients with neurological diseases, a pharmacist-led educational intervention markedly enhanced the prescription pattern and usage for sodium valproate, medication adherence, and disease control.

**Supplementary Information:**

The online version contains supplementary material available at 10.1186/s12913-025-12483-5.

## Introduction

Epilepsy is a chronic disease requires long-term care. Medication adherence is essential for managing symptoms and preventing seizures. Pharmacists play a crucial role in the care of epileptic patients by counselling and educating them on their illness, the appropriate medication use, and the importance of adherence [1]. The use of antidepressant and antipsychotic drugs to treat mental illness has transformed during the last 10 years. Patients on selective serotonin reuptake inhibitors may experience fewer anticholinergic side effects, dizziness problems, and cardiovascular issues than those on older tricyclic antidepressants [2].

To help patients receive the best pharmacological therapy, clinical pharmacists with experience in mental health can limit the undesirable drug-drug interactions, and the unnecessary use of multiple medications particularly with regard to appropriate drug selection and monitoring [3]. A successful implementation of the Slovenian model for ambulatory clinical pharmacists in primary care settings (Pharmacist Consultant) found a favorable effect on several pharmacotherapy measures and benefits, especially for patients with multiple diseases and medications [4]. Children with epilepsy who get active pharmacy education are more likely to adhere to their valproic acid therapy, according to a prior study [5]. So, pharmacist intervention can therefore enhance seizure management.

When it comes to managing recurrent seizures, noncompliance raises the risk of major clinical outcomes, such as increased mortality, and places a significant financial burden on the healthcare system [6]. Since medication adherence is crucial to the management of epilepsy, pharmacists are in a unique position to significantly impact the clinical outcomes [7, 8].

All over the world, individuals with psychotic diseases are urged to self-report adverse drug reactions (ADRs) [9]. Pharmacists pay attention to drug information provided for patients [10], from approved indications [11] to recommended dosages [12–15] and antipsychotic polypharmacy rates [16]. People with psychotic disorders often have notable use of multiple drugs which increases the risk of undesirable drug-drug interactions and adverse drug reactions (ADRs) [15, 16].

Medication use indicators are used to evaluate patient care, prescription trends, and facility-specific factors related to appropriate medication use [17, 18]. Drug use indicators have also been used to assess how often people with mental health disorders, who are in hospitals and those who live in the community, take psychiatric drugs [19, 20].

Sodium valproate is a broad-spectrum anti-seizure medication that regulates ɣ-aminobutyric acid to treat either generalized or focal seizures. Additionally, it affects serotonin, dopamine, and glutamate. This drug comes in a range of dosage types and is both affordable and effective. In the liver, valproate is extensively metabolized, mostly by glucuronidation, and then it goes through a complicated oxidation process that involves several cytochrome P450 enzyme systems. The medication generates several metabolites that might contribute to its possible toxicity as well as its therapeutic benefits [21].

Sodium valproate has several potential side effects and medication interactions. Thrombocytopenia, hepatotoxicity, and hyperammonemia are the main side effects of sodium valproate at lower doses [22]. For the greatest clinical outcomes, several screening and laboratory procedures must be followed prior to beginning sodium valproate treatment. The wise utilization of sodium valproate still needs additional regulatory measures, such as drug usage evaluation and pharmacovigilance reports [23].

Patients with neurological illnesses attending outpatient clinics at a tertiary care hospital were enrolled in this study, to assess the effects of pharmacist educational intervention and counseling on sodium valproate prescribing patterns and usage as well as medication adherence. The primary outcomes, includes an improvement in valproate prescription patterns and usage. The secondary outcome was improving patients’ adherence.

## Methods and study design

The present study is a quasi-experimental pretest–posttest design study that was performed at the Neurology Department outpatient pharmacy and clinics at Mansoura University Hospital (tertiary care hospital), Egypt. The study protocol was approved by the local ethical committee of Tanta University's Faculty of Pharmacy in Egypt and verified to comply with the Declaration of Helsinki's ethical guidelines (Code number: TP/RE/2/23p-006) on 2/01/2023. This study was registered in the National ClinicalTrials.gov with Code No: NCT05830981 on 25th April 2023).

### Eligibility criteria

**Fig. 1:**
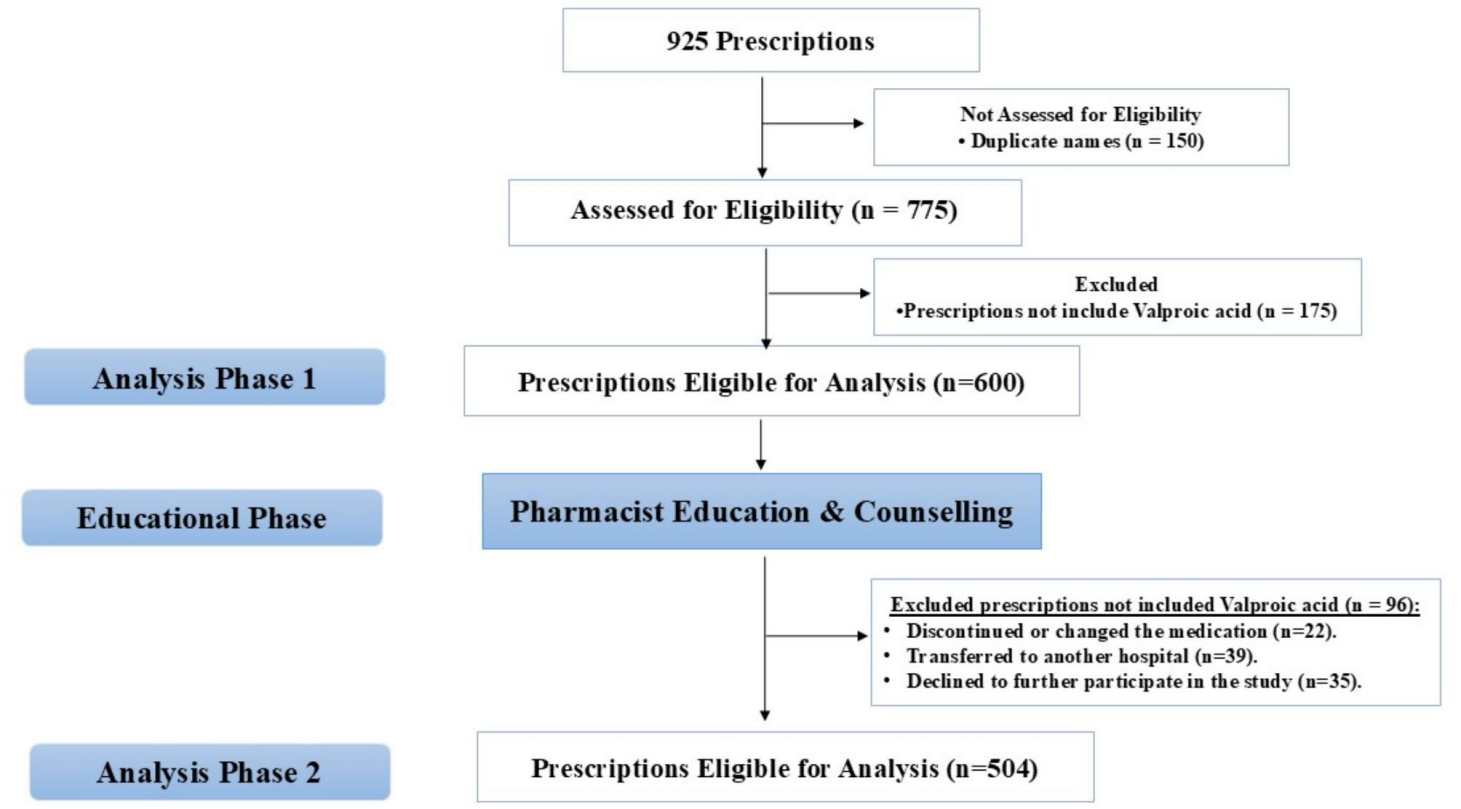
Study Flow Diagram

Patients with psychotic disorders who were treated with sodium valproate for at least seven days and who were continuously eligible for the experiment were included in the study. Selected prescriptions were from both sexes and patients were ranging in age from 10 to 70. Exclusion criteria include prescriptions for patients who were not on sodium valproate or who had other conditions like progressive myoclonus epilepsy, cortical development abnormalities, hippocampus sclerosis, tumors, chronic liver disease, chronic kidney disease, previous neurosurgery, developmental, or epileptic encephalopathy (Fig. 1).

### Study design

Using a quasi-experimental pretest–posttest approach, pharmacists evaluated the indicators and prescription patterns of sodium valproate in patients with neurological diseases. Between January 2015 and December 2017, pharmacists reviewed 925 prescriptions, selecting 600 for phase one evaluation (Fig. 1). Patients who fulfilled the eligibility criteria were enrolled by the pharmacists to participate in the study, and all patients or their parents/guardians provided their informed permission at the time of the patient's first visit to the pharmacy clinic.

A well-structured form was used to collect the required patient data on a spreadsheet. Along with other prescription data (patients' original therapeutic indications, concurrent usage of oral drugs, major adverse drug effects, and any drug-drug interactions), collected data also contained patient demographics (gender, age, weight, and creatinine clearance) [24].

Following a three-month of follow-up and counseling educational intervention, hospital clinical pharmacists reevaluated the patients' prescriptions (phase 2). The recommended doses for adults were as follows: for adjunctive therapy or initial monotherapy or complex partial seizures, 10–15 mg/kg/day orally of sodium valproate, or 15 mg/kg/day orally for absence seizures; increased at weekly intervals by 5 to 10 mg/kg/day until optimal therapeutic levels and/or clinical response reached. The maximum suggested dose was 60 mg/kg/day [25]. The recommended doses of pediatrics were as follows: initially, in 1 to 3 divided doses, 10 to 15 mg/kg/day; then increased by 5 to 10 mg/kg/day until seizures are controlled, or side effects prevent additional increases at weekly intervals. The daily doses > 250 mg must be administered in 2 to 3 divided doses; for maintenance, 30 to 60 mg/kg/day. However, Children undergoing polytherapy, or receiving more than one anti-seizure, may need dosages of up to 100 mg/kg/day divided into three or four doses [26].

### Valproate prescribing pattern indicators

1. *Maximum-dose and Minimum-dose Sodium Valproate*: The percentage of patients prescribed antipsychotic medicine, either one type or a mix, at a daily dose exceeding the recommended amount in the Therapeutic Guidelines [27]. For each medicine, the suggested amount was changed into a percentage of the highest allowed amount. Then, all these percentages were added together to get the overall daily amount [28, 29].
2. *Antipsychotic Combination:* The number of patients who are given two or more medicines from the same drug category at the same time.
3. *Low doses of mood stabilizers*: Percentage of patients prescribed daily dose of sodium valproate < 1000 mg or carbamazepine < 600 mg.
4. *Tolerability:* How well patients handled the treatment was checked by looking at any bad effects written down in their medical records.
5. *Patient Adherence Assessment*: The Simplified Medication Adherence Questionnaire (SMAQ) was the tool used to check if people were taking their medicine as they should [30]. The English SMAQ was turned into an Arabic version with both open-ended and number-based questions. Patients received it whenever they got their prescriptions refilled. The qualitative questions comprise the following: (1) “Have you ever missed a dose of your medication?”; (2) “Are you careless at times about taking your medicine?”; (3) “Do you ever stop taking your medicine when you start feeling better?”; (4) “If you feel worse, do you stop taking your medicine?”; and (6) “Did you skip taking your medicine at all during the previous weekend?”. The quantitative questions involve the following: (5) “In the past week, how often did you not take your medicine?”; and (7) “Over the past 3 months, how many days have you not taken any medicine at all?”. A patient was considered not following their treatment plan if they answered "yes" to any of questions 1, 2, 3, 4, or 6; or if they skipped their medicine for more than two days in the last three months; or if they missed more than two doses in the past week.
6. *Beneficial Effect:* is the total seizure control without having undesirable side effects [31]. Good control is defined as not having any seizures since the last appointment, while fair control is defined as having one seizure since the last appointment, whereas poor control is defined as having many seizures since the last appointment.

### Strategies to improve valproate prescribing pattern and patients’ adherence

**Fig. 2:**
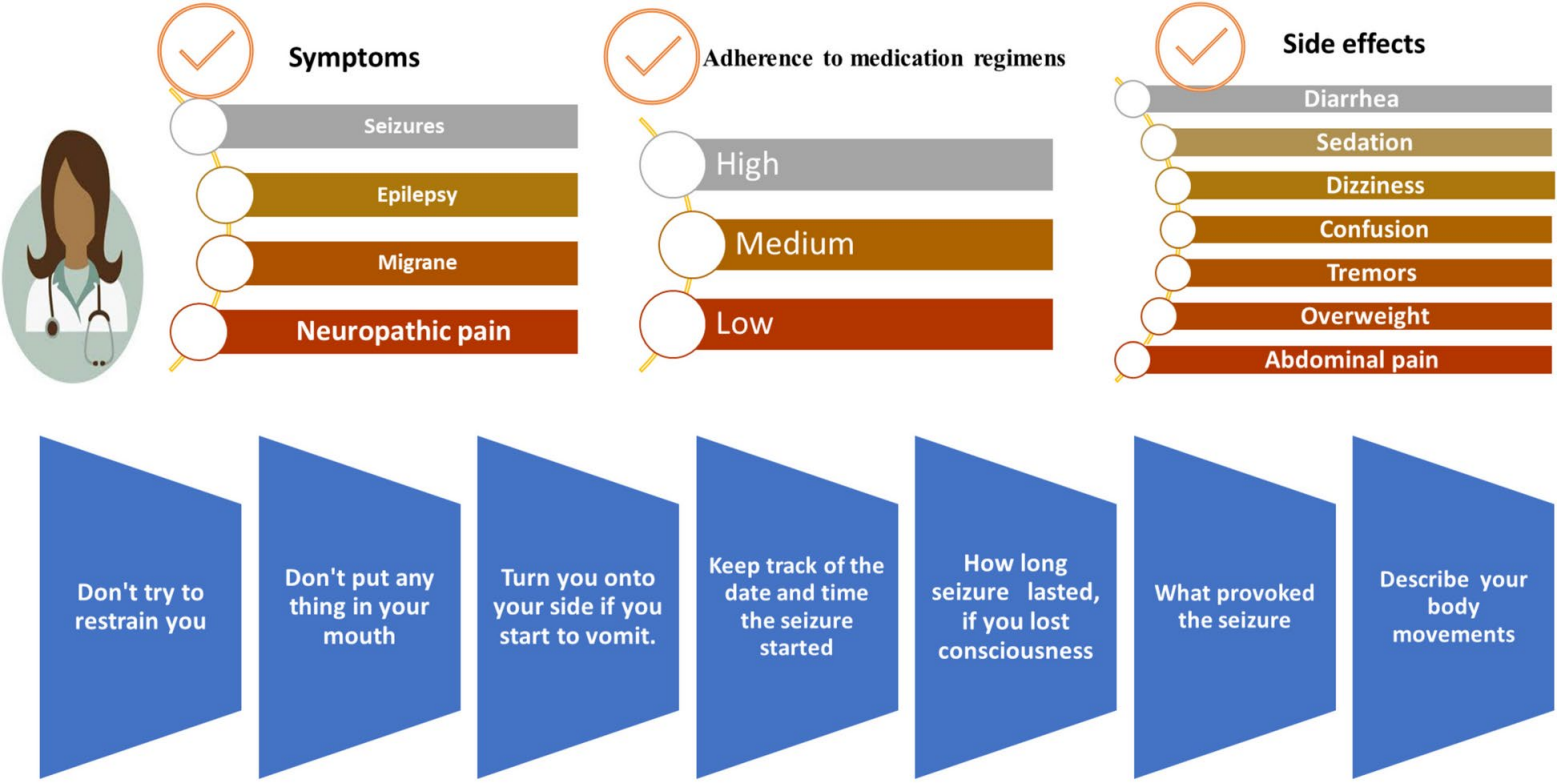
The educational printed leaflet and patient counselling

Neurologists and pharmacists worked together to customize patient counseling [32]. After a follow-up, patients whose prescriptions were chosen received educational interventions from clinical pharmacists in the neurology department's outpatient pharmacy in the form of lectures, information bulletins, audits, and feedback procedures. Patients received counseling on the objectives of anti-seizure therapy and the necessity of strict adherence to these objectives. In addition, instructions on medicine administration and a prescription regimen were offered to meet the patient's daily routines, emphasizing the need to attend necessary clinic sessions for planned follow-ups to encourage adherence. The average length of the educational and counseling session was thirty minutes. Patients were given a copy of an educational printed booklet (Fig. 2) to aid with the education program and the patient was given an Arabic copy to take home. During the study, patients or their family members received the pharmacist's educational intervention at the hospital out-patients pharmacy [32].

### Prescription refill and patients’ adherence

Each patient calculated the expected dates for ordering more medicine supplies using a prescription record book. Patients were reminded of their next clinic visits by phone calls or colorful cards if they had not visited the hospital pharmacy in the time specified. Techniques for increasing adherence such as medication diaries, pill containers, patient counseling, phone calls for prescription refills, and appointment reminders, were used.

### Statistical analysis

The collected data was recorded on an electronic spreadsheet. Statistical Product and Service Solutions (SPSS) (version 21.0) were used for Statistical analyses [33]. G*Power software version 3.1.0 was used for sample size calculation. It was estimated that a total sample size of 500 prescriptions would have a power of 99% to detect a medium to large effect size of 0.40 in the outcome measure. Cronbach's α was used to measure the reliability of the SMAQ. Qualitative data were presented as numbers and percentages and were compared using Pearson's chi-square test or Fisher's exact test. Continuous variables were expressed as mean ± standard deviation and compared by the student’s *t*-test. All *p* values were two-tailed and *p* < 0.05 was considered statistically significant.

## Results

Nine-hundred and twenty-five patients’ prescriptions at the Neurology Department outpatient pharmacy were reviewed during the study in phase one. 600 prescriptions containing anti-seizure sodium valproate were analyzed at baseline before educational intervention. Table 1 displays the demographic data, medical history, creatinine clearance, and indication of valproate use. Collected data showed that female patients made up 55% (*N* = 330), while male patients made up 45% (*N* = 270) of the total enrolled prescriptions. The age range of most participants was 20–39 years (59.7%; *n* = 358), followed by 40–69 years (22.8%; *n* = 137) and 10–19 years (17.5%; *n* = 105) (Table 1). Data collected after education intervention showed that most of the participants aged between 20 and 39 years old (59.9%, *n* = 302), then between 40 and 69 years old (22.8%, *n* = 115). However, the age range was 31.30 ± 12.34 years vs 31.48 ± 12.40 years for enrolled patients before vs after education intervention (Table 1).

**Table 1:** Patients' demographic and clinical characteristics before and after the educational intervention

**Data**	**Number of prescriptions Before** ***n*** = 600	**Number of prescriptions** **After** ***n*** = 504	***P*** Chi (2-sided)
**Age (years)**	31.30 ± 12.34	31.48 ± 12.40	0.79*
10-19y	105 (17.5)	87 (17.3)	0.80
20-39y	358 (59.7)	302 (59.9)	0.80
40-69y	137 (22.8)	115 (22.8)	0.80
**Sex**			
Male	270 (45)	224 (44.4)	0.85
Female	330 (55)	280 (55.6)	0.80
**Body weight**			
≥ 60 kg	376 (62.7)	312 (61.9)	0.80
< 60 kg	224 (37.3)	192 (38.1)	
**Creatinine clearance**			
≥ 95 mL/min	489 (81.5)	435 (86.3)	0.19
50‐95 mL/min	90 (15)	57 (11.3)	0.17
15‐50 mL/min	15 (2.5)	9 (1.8)	0.198
< 15 mL/min	0 (0)	0 (0)	-
No data	6 (1)	3 (0.6)	0.76
**Indications of use**			
Epilepsy	462 (77)	369 (73.2)	0.15
Bipolar disorder	138 (23)	135 (26.8)	0.17
Neuropathic pain	0	0	-
Migraine	0	0	-

Data were presented as mean ± SD (for normally distributed variables). Qualitative data were expressed as numbers (percentages)

Chi-square (χ2) test and Monte Carlo test were used for comparison between groups

*P* < 0.05 was considered significant

*Student’s *t*-test

Most of the included patients (*n* = 489, 81.5%) had normal creatinine clearance ≥ 95 mL/min. Ninety (15%) patients showed mild kidney dysfunction, and fifteen (2.5%) patients showed moderate to severe kidney dysfunction. Likewise, Table 1 displays that the patient's demographic data, medical history, and creatinine clearance for the prescriptions issued after pharmacist education; did not alter much from the analysed prescriptions before the educational intervention (*P* ≥ 0.05).

**Fig. 3:**
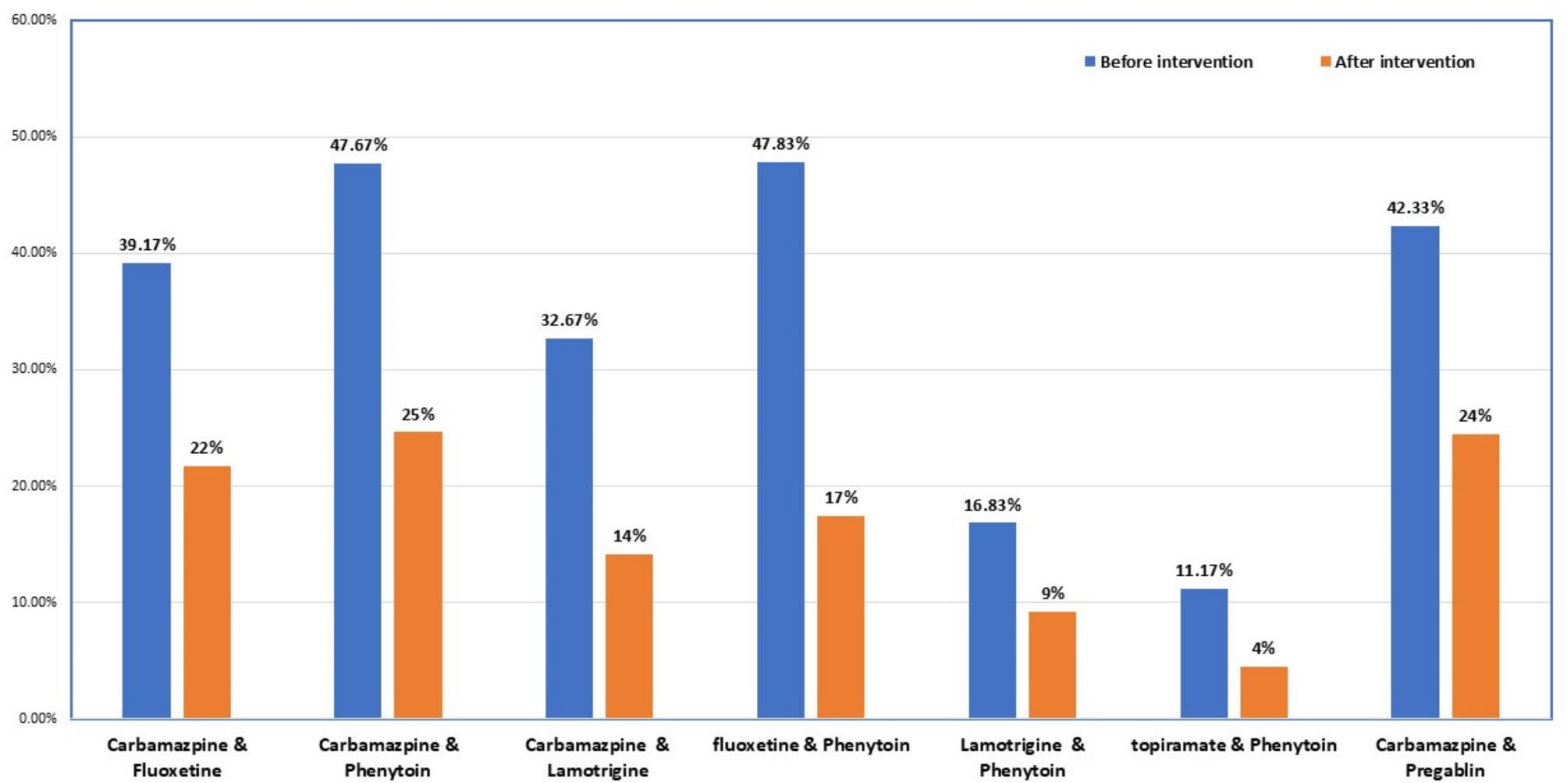
Percentage of anti-seizure and psychiatric drugs that prescribed with sodium valproate before and after the educational intervention

Figure 3 shows the percentage of anti-seizure and antipsychotic drugs that may interact with sodium valproate in the analyzed prescriptions before and after educational intervention. Both carbamazepine & fluoxetine were prescribed 235 (39.17%) *vs.* 111* (*22%), carbamazepine & phenytoin by 286 (47.67%) *vs*. 126 (25%), carbamazepine & lamotrigine by 196 (32.67%) *vs.* 71 (14%), fluoxetine & phenytoin by 287 (47.83%) *vs*. 86 (17%), lamotrigine and phenytoin by 101 (16.83%) *vs*. 46 (9%), topiramate & phenytoin by 67 (11.17%) *vs.* 20 (4%) and carbamazepine & pregabalin by 254 (42.33%) *vs.*121* (* 24%), before vs after educational intervention, respectively (Fig. 3).

**Fig. 4:**
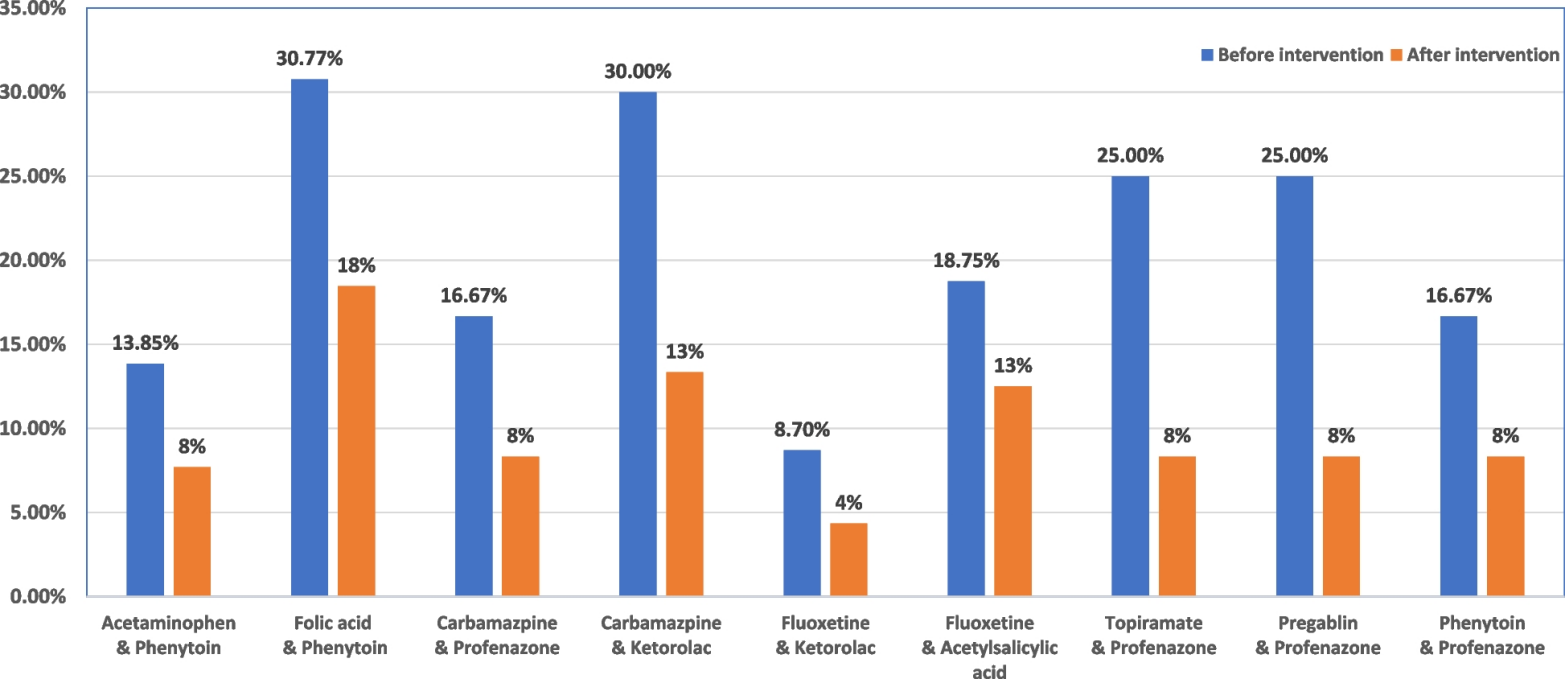
Percentage of non-psychiatric drug that may interact with sodium valproate before and after the educational intervention

Co-administration of two or more medications that raise the risk of weakness, dyskinesia, and extrapyramidal symptoms is an often-documented drug-drug interaction. The co-administration of ketorolac, folic acid, acetylsalicylic acid, acetaminophen, and profenazone that may result in non-psychiatric drug-drug interactions is shown in (Fig. 4).

Valproate prescription pattern indicators were displayed in Table 2 both prior to and after the educational intervention. Phase 2 included 504 prescriptions, 96 were excluded, they either discontinued or changed the medication (*n* = 22), or transferred to another hospital (*n* = 39) or declined to further participate in the study (*n* = 35) as shown in Fig. 1.

**Table 2:** Valproate prescribing pattern indicators before and after the educational intervention

Indicators	Number of prescriptions Before n = 600	Number of prescriptions After n = 504	*P* Chi (2-sided)	Fisher’s (2-sided)
Prescribed max-dose valproate	122 (20.3)	85 (16.9)	0.14	0.16
Prescribed min-dose valproate	137 (22.8)	27 (5.35)	< 0.001	< 0.001
antipsychotic combination ≥ 2 drugs from same therapeutic class	52 (8.67)	21 (4.2)	0.003	0.003
Prescribed antipsychotic drug	52 (8.67)	19 (3.8)	0.001	0.001
Low doses of mood stabilizers	31 (5.2)	28 (5.6)	0.78	0.79
Antipsychotic polypharmacy	43 (7.2)	16 (3.2)	0.003	0.003
**Number of drugs included in the prescriptions analyzed**				
One drug	69 (11.5)	51 (10.1)	0.64	0.67
Two drugs	329 (54.8)	262 (52)	0.6	0.7
Three drugs	169 (28.2)	163 (32.3)	0.64	0.67
Four drugs	18 (3)	16 (3.2)	0.7	0.74
Five drugs	15 (2.5)	12 (2.4)	0.87	0.88
**Medication errors regarding frequency and dose of Sodium valproate**				
Frequency	29 (4.8)	8 (1.6)	0.003	0.004
Doses	35 (5.8)	6 (1.2)	< 0.001	< 0.001
**Contraindications**				
Pregnancy, breastfeeding:	0 (0)	0 (0)	-	-
hepatic failure	0 (0)	0 (0)	-	-

Qualitative data were expressed as numbers (percentages)

Chi-square (χ2) test and Monte Carlo test were used for comparison between groups

*P* < 0.05 was considered significant

The sodium valproate frequency or prescribed dose errors compared to baseline were improved significantly after the educational intervention by 29 (4.83%) *vs* 8 (1.6%) (*p* = 0.004) and 35 (5.8%) *vs.* 6 (1.2%) (*p* = 0.00) of theprescriptions, respectively (Table 2).

Eight drug use indicators were used before and after the educational intervention to assess the psychiatric drug use pattern as the following: Prescribed high-dose antipsychotics were found in 122 (20.33%) *vs* 85 (16.9%) prescriptions. The prescribed minimal dose was 137 (22.8%) vs 27 (5.35%) (*p* = 0.000) of the prescriptions. However, receiving an antipsychotic combination was found in 43 (7.2%) *vs* 16 (3.2%) (*p* = 0.003) of prescriptions. On the other hand, no patient was discovered using three psychiatric medicines at the same time. Moreover, prescriptions with antipsychotic combination ≥ 2 from the same therapeutic class were 52 (8.67%) *vs 21 (*4.2%) (*p* = 0.003) (Table 2).

The findings demonstrated that anti-seizure medication adherence was considerably raised by a combination of adherence-improving strategies. As a result, the average frequency of seizures decreased in half. The data from (Table 3) found that 292 (57.93%) patients were in good control *vs.* 185 (30.8%) patients (*p* = 0.000). However, patients in fair control were 184 (36.5%) *vs*. 356 (59.3%) (*p* = 0.000) and 28 (5.5%) patients *vs.* 59 (9.8%) were in poor control (*p* = 0.000).

**Table 3:** Effect of the educational intervention on the patient’s safety and adherence

Criteria	Number of prescriptions Before *n* = 600	Number of prescriptions After *n* = 504	P Chi (2-sided)
**Adherence to medication regimens**			
High	50 (8.3)	338 (67.1)	< 0.001
Medium	492 (82.0)	150 (29.8)	< 0.001
Low	58 (9.7)	16 (3.2)	< 0.001
**Side effects**			
Nausea	7 (1.2)	3 (0.6)	0.32
Diarrhea	5 (0.8)	3 (0.6)	0.64
Weight gain	8 (1.3)	6 (1.2)	0.83
Abnormal liver function,	6 (1)	2 (0.4)	0.24
Fatigue, sedation, confusion, and dizziness	30 (5)	14 (2.8)	0.06
Tremor	2 (0.3)	1 (0.2)	0.17
**Beneficial effect**			
Good control	185 (30.8)	292 (57.9)	< 0.001
Fair control	356 (59.3)	184 (36.5)	< 0.001
Poor control	59 (9.8)	28 (5.6)	< 0.001

Qualitative data were expressed as numbers (percentages)

Chi-square (χ2) test and Monte Carlo test were used for comparison between groups.

*P* < 0.05 was considered significant

Weight gain, diarrhea, irregular liver function, nausea, and upper abdominal cramps are common side effects of valproate. Tremor, fatigue, drowsiness, disorientation, and dizziness were among the common adverse neurological symptoms that eventually disappeared. However, following the educational intervention, non-significant improvements in side effects were noted (Table 3).

## Discussion

The current study aimed to assess the prescribing pattern and usage of sodium valproate among patients enrolled from an outpatient pharmacy of a tertiary care psychiatric facility in Egypt. Assessed valproate prescriptions showed that most of the patients diagnosed with epilepsy and bipolar disorder.

The demographic characteristic of patients in the current study revealed that women with epilepsy were more prevalent than men. The age group of 20 to 39 years old was shown to have the highest frequency of neurological diseases. Here, we discovered that the most often prescribed medicine classes were mood stabilizers, antidepressants, and anti-seizure medications. The antipsychotic combination after pharmacist education was around 3.2, higher than the 1.79 to 2.5 drugs per prescription observed in the previous similar studies [34, 35].

Furthermore, the current study showed that antipsychotic combinations were inevitable, which means they could lead to medication errors, drug interactions, adverse drug reactions, underuse, or elevated serum levels of effective treatments. These outcomes can raise the risk of chronic toxicity, which includes neurocognitive issues. It is linked to increased prescription costs and the need for therapeutic drug monitoring, and it may also have an impact on adherence. However, in people with epilepsy, antipsychotic combinations could be the sole means of improving seizure control [36].

In the current study, about 87.5% of the 600 prescriptions that were analyzed prescribed more than one medicine, and 2.5% prescribed five drugs. Several facts demonstrate that proper polypharmacy is required in some illnesses and situations [16]. However, prior study that showed that instructions of psychiatrists and nurses may reduce the use of multiple medications. Further decreases were associated with the inclusion of psychiatrists who got monthly audit feedback [37].

Sodium valproate was the most frequently prescribed anti-epileptic drug (AED), followed by carbamazepine for management of all types of generalized seizures. On the other hand in partial seizure, the most frequently prescribed AED was carbamazepine followed by phenytoin [38]. Although medical practitioners may not have specific training in recognizing different seizure types and epileptic syndromes, AEDs should provide a variety of activities that are easy for them to manage [39].

Medical professionals at government tertiary care centers were not required by local health administration directives to prescribe generic names, which is against WHO guidelines, as generic name prescriptions are a sign of rational prescribing [40].

To elicit a response in bipolar disorder, a sodium valproate dose of at least 1000 mg per day and a serum concentration of 50 mg/L are usually required [41]. Elevated plasma concentrations of psychiatric drugs may result from sodium valproate's ability to block the metabolism of all types of these drugs [42]. It is thought that a blood level of at least 7 mg/L and a daily dosage of 600 mg of carbamazepine are necessary for a clinical response in affective disorders [43]. CYP3A4 metabolizes several psychiatric drugs and is known to be activated by carbamazepine [44]. This may result in lower plasma concentrations of CYP3A4 substrate medications taken simultaneously; as a result, increasing the dosage may be necessary to get the desired therapeutic effect. However, discontinuing carbamazepine suddenly may make it more likely that other mental drugs' plasma concentrations would rise unexpectedly. When medications are used inappropriately, the patient wastes resources and has negative drug responses that hurt them and result in poor patient outcomes [45].

Pharmacists' regular evaluations of prescription regimens may help identify potential drug-related problems in patients with mental illnesses [45]. Furthermore, it's critical to inform patients and their caregivers about the symptoms of common adverse medication reactions (ADR) [46]. ADR underreporting is a major and widespread public health problem, and it may be particularly true for ADRs associated with mental diseases [47]. Patients and their caregivers should be included in decisions regarding mental medicine regimes. This is especially true when prescripted drugs are given in combinations and quantities that deviate from accepted therapeutic guidelines. However, patients' knowledge and perceptions of the factors influencing treatment regimen selection were not examined in the current investigation.

Previous research indicates that pharmacists significantly improve patients' medication adherence, which influences epilepsy treatment [48]. According to their disease management, the current study found that pharmacist-led educational interviews enhanced medication adherence in individuals with epilepsy.

Additionally, the current study discovered that an educational intervention on the frequency and dosage of sodium valproate enhanced patient adherence to treatment. Consistently, for improved patient adherence, a combination of simple strategies, such as patient counseling and customized drug containers, would be more successful than either strategy alone [49]. Medication adherence has been shown to increase by self-recording medication intake, prescription refill reminders, and tailoring medication use to daily habits [50].

Active pharmacist assistance is crucial for dementia patients, according to Nanaumi et al. (2022), not only to guarantee proper medication management but also to possibly enhance patient and caregiver quality of life by reducing the burden of care [51]. Continuous pharmacist participation improved medication adherence and therapeutic valproic acid use in children epileptic patients [1]. Improved patient adherence and authorization in the patients' pharmaceutical care have resulted from pharmacists' specialized counseling, which has raised caregivers' knowledge and comprehension of epilepsy and AED use. This has also given them more confidence when prescribing and administering AEDs [52].

Contrary to previous studies [53], the postal appointment reminder cards use did not improve pharmacy appointment attendance in the current study. Our results, however, are not unexpected as we found that the two main obstacles to maintaining appointments are asking for time off from work and having transportation problems, neither of which is resolved by sending reminders. Therefore, it would probably be more beneficial to provide more flexible clinic hours (such as an evening appointment at the epilepsy pharmacy) to boost planned appointment attendance.

The prevalence of drug-related problems found in a recent study highlights the clinical role of pharmacists and the importance of interprofessional collaboration in the care of neurological patients, especially in the pharmaceutical follow-up of elderly individuals, given the importance of preventing and resolving drug-related issues in healthcare settings [54].

Likewise, another study highlights how important it is to recognize and avoid drug-related problems in people with neurological disorders. A unit-based clinical pharmacist's active involvement in treatments and their high acceptance rate by other medical professionals lead to better patient care and more reasonable pharmaceutical prescriptions [55]. Following up patients with pharmacists who specialize in neurological and mental diseases improves the results for patients with these conditions [56].

Despite warnings, women and adolescents of reproductive age are nevertheless administered valproic acid. In women and girls, valproic acid increases the risk of obesity, hormone disruption, and polycystic ovarian syndrome (PCOS) [57].

Recent research has shown valproic acid's teratogenicity and ability to alter hormones. Exposure to valproic acid during pregnancy increases the chance of various severe birth abnormalities, lowered IQ, and behavioral problems. VPA usage recommendations are still not widely followed by women and girls of reproductive age, despite the significant and well-established risk [57].

To better grasp various issues associated with women's health in epilepsy, pharmacists can benefit from increased exposure to and interaction with people who have the disease, even if they may be knowledgeable and in a unique position to provide patients with chronic illnesses with important information [58].

Epilepsy is a suitable target for drug adherence study since it is a chronic, often lifelong disorder with a reasonably frequent and measurable primary outcome (seizures) [59]. If epilepsy medications are not taken as prescribed, there might be major consequences. Nevertheless, despite the dangers of non-compliance, previous studies have shown that only around 75% of people with epilepsy follow their anti-seizure drug regimens [60, 61]. Ernst et al. found that women reported higher percentages of adherence to anti-seizure medicines than to other prescription drugs and supplements. This implies that the perceived value of therapies probably affects prescription adherence, which calls for more study [62].

These findings demonstrate the critical role of clinical pharmacists who specialized in mental or neurological conditions in providing those patients safe, and effective care. Documenting patient-level outcomes should be the primary focus of psychiatric pharmacists' future efforts to create and offer innovative or creative clinical pharmaceutical services.

## Limitations

The study was short length, non-RCT design, single-center, small sample size, and a few months follow up after the intervention restricted the time frame over which effects could be seen. Additionally, patient groups in other hospitals or with different diagnostic profiles or comorbidities may not be well represented by the patient population we analyzed. Therefore, it is necessary to conduct a longer and larger sample-size multi-center research in the future.

## Conclusion

More reasonable prescribing practices should be used, and sodium-valproate dosage and frequency should be routinely checked. This study demonstrated that in epileptic patients, a set of low-cost, readily implemented strategies, including pharmacist educational intervention, significantly improved medication adherence and clinical control of seizure, thus lowering disability and social handicaps caused by epilepsy. This study offers baseline data for further research to assess the pharmacist impact on prescribing practices in tertiary care units, which may provide insights for the most effective use of neurological medications in mental health facilities to improve patient's health and quality of life.

## Supplementary Information

Supplementary Material 1

## Data Availability

The corresponding author can provide the data supporting the study’s conclusions upon reasonable request.
